# Catalysts of DNA Strand Cleavage at Apurinic/Apyrimidinic Sites

**DOI:** 10.1038/srep28894

**Published:** 2016-07-01

**Authors:** Irina G. Minko, Aaron C. Jacobs, Arnie R. de Leon, Francesca Gruppi, Nathan Donley, Thomas M. Harris, Carmelo J. Rizzo, Amanda K. McCullough, R. Stephen Lloyd

**Affiliations:** 1 Oregon Institute of Occupational Health Sciences, Oregon Health & Science University, Portland, Oregon 97239, United States; 2 Department of Chemistry, Vanderbilt University, Nashville, Tennessee 37235, United States; 3 Department of Molecular and Medical Genetics, Oregon Health & Science University, Portland, Oregon 97239, United States; 4 Department of Physiology and Pharmacology, Oregon Health & Science University, Portland, Oregon 97239, United States

## Abstract

Apurinic/apyrimidinic (AP) sites are constantly formed in cellular DNA due to instability of the glycosidic bond, particularly at purines and various oxidized, alkylated, or otherwise damaged nucleobases. AP sites are also generated by DNA glycosylases that initiate DNA base excision repair. These lesions represent a significant block to DNA replication and are extremely mutagenic. Some DNA glycosylases possess AP lyase activities that nick the DNA strand at the deoxyribose moiety via a β- or β,δ-elimination reaction. Various amines can incise AP sites via a similar mechanism, but this non-enzymatic cleavage typically requires high reagent concentrations. Herein, we describe a new class of small molecules that function at low micromolar concentrations as both β- and β,δ-elimination catalysts at AP sites. Structure-activity relationships have established several characteristics that appear to be necessary for the formation of an iminium ion intermediate that self-catalyzes the elimination at the deoxyribose ring.

Apurinic/apyrimidinic (AP) sites are common DNA lesions that occur naturally through deglycosylation of DNA, loss of unstable modified nucleobases, or as an intermediate in the base excision repair (BER) pathway^1^,^2^,^3^,^4^. If left unrepaired, AP sites can inhibit DNA replication and transcription and contribute to mutagenesis^5^,^6^,^7^,^8^. In the BER pathway, AP sites are generated by DNA glycosylases that hydrolyze the bond between the damaged base and the deoxyribose^1^,^3^,^9^. The newly-formed AP sites can be further processed by an AP endonuclease that cleaves the phosphodiester DNA backbone on the 5′ side of AP sites^2^,^10^. This creates a 3′-hydroxyl to enable initiation of gap-filling DNA repair synthesis.

Many DNA glycosylases, including human NEIL1, NEIL2, NEIL3, NTH1 and OGG1, possess AP lyase activities that catalyze either a β- or β,δ-elimination reaction at the deoxyribose moiety^9^,^11^,^12^,^13^,^14^. This reaction proceeds via the formation of a transient enzyme-DNA covalent intermediate and generates DNA breaks with a phospho-α, β-unsaturated aldehyde or phosphate group at the 3′-end. In addition to these enzyme-catalyzed reactions, non-enzymatic cleavage of DNA at AP sites occurs under alkaline conditions or elevated temperatures^1^,^15^,^16^ and in the presence of polyamines^9^,^15^,^17^,^18^,^19^, histones^9^,^20^, various nucleophilic peptides^21^,^22^,^23^, or photoactivated metalloinserters^24^. For many of these compounds, the strand scission has been shown to proceed through a β-elimination of an imine intermediate and is inhibited by the presence of methoxyamine^25^.

Due to the biologically significant role of AP sites, previous efforts have been made to engineer effective non-enzymatic AP lyase catalysts, such as the acridine-nucleobase dimers linked by a polyamine chain^26^. Although these so-called “artificial nucleases” appeared to be potent in cleavage of AP site-containing DNA *in vitro*, no reagents or drugs have been developed based on this design for use *in vivo*. Thus, the availability of small molecules that possess AP lyase activity remains largely an unmet need.

In conjunction with our investigations to identify inhibitors of DNA glycosylases, screens of small molecule libraries revealed that hNEIL1 was inhibited by several purine analoques^27^. To potentially expand the number of lead compounds, a library of selected kinase inhibitors^28^ (courtesy of F. Hoffmann-La Roche Inc., Dr. Paul Gillespie) was assayed as inhibitors of hNEIL1 or hOGG1, since many of these small molecules are composed of a core purine-like structure. Although no effective inhibition of either glycosylase was observed, several of these compounds showed an unexpected ability to efficiently cleave AP site-containing DNA. Herein we describe structure-activity and mechanistic studies of this new class of small molecule catalysts for DNA strand incision.

## Results and Discussion

### Compound C1 can cleave DNA containing AP site

**Figure 1:**
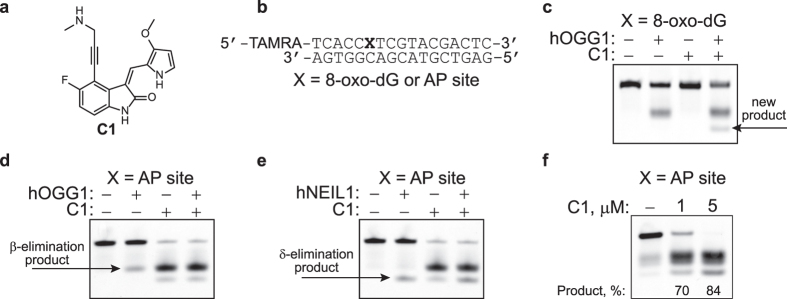
Non-enzymatic DNA cleavage at an AP site. AP site-containing DNA was obtained by treating the corresponding dU-containing DNA with UDG. **(a)** Structure of a representative catalyst for DNA scission. **(b)** DNA substrates. **(c)** Scission of 8-oxo-dG-containing DNA (250 nM) in the presence of hOGG1 (100 nM) and **C1** (10 μM). **(d)** Scission of AP site-containing DNA (250 nM) in the presence of hOGG1 (50 nM) and **C1** (10 μM). **(e)** Scission of AP site-containing DNA (250 nM) in the presence of hNEIL1 (50 nM) and **C1** (10 μM). **(f)** Scission of AP site-containing DNA (2 μM) by **C1**. The percent of products was corrected for the spontaneous cleavage. Reactions were carried out at 37 °C for 30 min (**b**–**d**) or 16 h **(e)**.

The initial observation that suggested the presence of an unusual activity in a subset of the small molecules under study was made when **C1** (Fig. 1a) was tested as a potential inhibitor of hOGG1. Specifically, addition of this compound to reactions of hOGG1 with a fluorescently-labeled duplex oligodeoxynucleotide containing a site-specific 8-oxo-dG adduct (Fig. 1b) resulted in an additional product that migrated faster than the β-elimination product; hOGG1 alone yielded only the β-elimination product (Fig. 1c). No nicked products were observed when 8-oxo-dG-containing DNA was incubated with **C1** alone.

Several mechanistic possibilities were hypothesized that could account for the formation of the new product. 1) In the presence of **C1**, hOGG1 had acquired the ability to catalyze a δ-elimination reaction in addition to its glycosylase and β-elimination AP lyase functions; 2) **C1** could convert the β-elimination product to the δ-elimination product; and 3) AP sites formed in the glycosylase reaction, could serve as a substrate for a **C1**-catalyzed β,δ-elimination reaction. To address these possibilities, duplex DNA containing a single site-specific AP site was generated by treating the corresponding dU-containing DNA with uracil DNA glycosylase (UDG) (Fig. 1b) and reacted with **C1** or hOGG1, either individually or in combination. As with the 8-oxo-dG-containing oligodeoxynucleotide, hOGG1 produced the expected β-elimination product, while the addition of **C1** produced a mixture of two products (Fig. 1d). This product mixture was also formed when the AP-containing oligodeoxynucleotide was incubated with **C1** alone. While the position of the slower migrating product band corresponded to the β-elimination product of hOGG1 (Fig. 1d), the faster migrating product band co-migrated with the known δ-elimination product of hNEIL1 (Fig. 1e). Thus, **C1** promotes the β- and δ-elimination reactions at AP sites.

The cleavage reaction by **C1** on AP site-containing DNA was concentration dependent (Fig. 1e and Supplementary Figure S1), with low micromolar concentrations being sufficient to observe the products. Similar to the previously designed “artificial nucleases”^26^, **C1** showed turn-over catalysis on the DNA substrate: 1 pmol **C1** generated ~1.4 pmol products in 16 h (Fig. 1f). Thus, the rate of incision by **C1** on this DNA was ~1.5 × 10^−3^ min^−1^ or higher. The comparison of **C1** with the currently available AP lyase reagents spermine^17^ and the KWKK peptide^23^ demonstrated that the newly-identified catalyst was at least 100-fold more efficient (Supplementary Figure S1).

### Several structural moieties in C1 are important for catalysis

**Figure 2:**
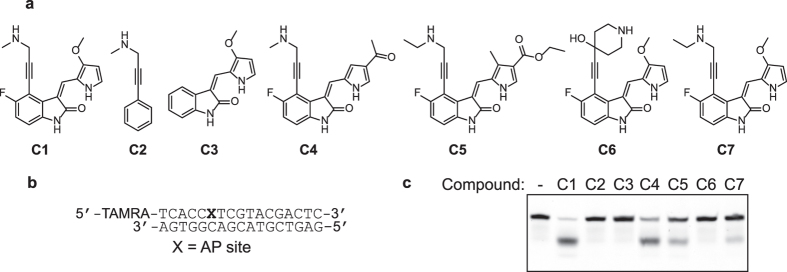
Structure-function analyses of the catalysts for DNA scission at an AP site. **(a)** Structures of representative compounds. **(b)** DNA substrate. **(c)** Assay for the abilities of representative compounds (10 μM) to incise AP site-containing DNA (250 nM). Reactions were carried out at 37 °C for 30  in.

Analogues of **C1** were assayed to determine the contribution of specific structural features to the strand scission chemistry. We initially examined the ability of simple, commercially available amines lacking the indolinone-pyrrole moiety **C2** (Fig. 2) and **CS1**-**CS3** (Supplementary Figure S2) to incise DNA at an AP site. Formation of incision products, if any, was below the level of detection at 10 μM. Thus, the indolinone-pyrrole moiety is important for the reaction. It is possible that the indolinone-pyrrole subunit binds to the AP site^29^ or minor groove and positions the secondary amine to catalyze strand cleavage via a covalent intermediate with the AP site. Compounds **C3** (Fig. 2) and **CS4** (Supplementary Figure S2) that contained the indolinone-pyrrole moiety but lacked the secondary amine were also completely inactive. These data strongly suggested the role for the amine as the reactive functional group. The replacement of the methoxy group on the pyrrole by other substitutions, such as in **C4** and **C5** (Fig. 2), led to decreased activity. The reaction was also modulated by a substituent on the amino group, such that increasing the steric demands of the secondary amine decreased the amount of the product observed (compare **C1**, **C6**, and **C7** (Fig. 2), and **C4**, **CS5**, and **CS6** (Supplementary Figure S2)). These structure-activity analyses demonstrated that several of the individual moieties are necessary but not sufficient for cleavage, and that these moieties must act cooperatively to produce strand scission.

### DNA structure modulates the C1-catalyzed cleavage

**Figure 3:**
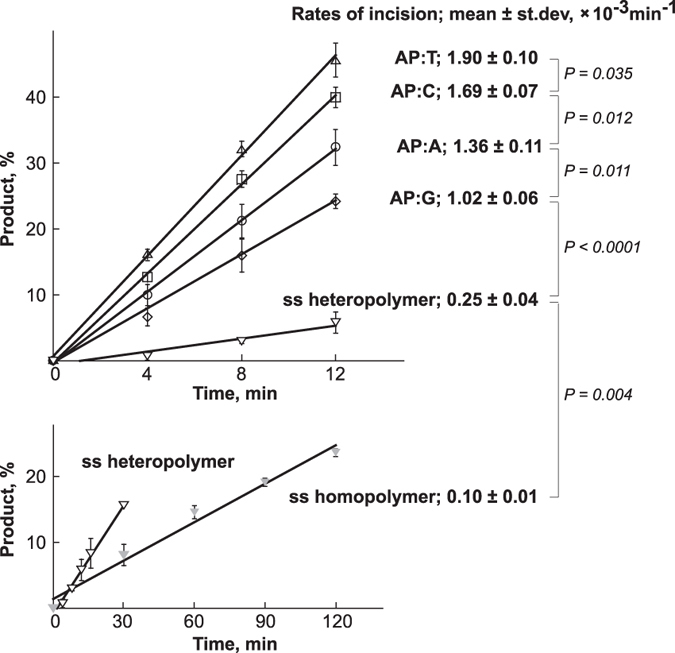
Rates of **C1**-catalyzed cleavage of AP site-containing DNA. Reactions were carried out at 37 °C using 250 nM DNA and 5 μM **C1**. The mean initial rates with respective standard deviations were calculated from three independent experiments using KaleidaGraph 4.1 software (Synergy Software). The P values were calculated using Students’ *t*-test.

The importance of the indolinone-pyrrole moiety for the strand scission chemistry has suggested that the structure of the DNA substrate could affect the **C1**-mediated cleavage. To address these relationships, the initial rates of reaction were measured for a single-stranded DNA (the sequence as in Fig. 1a) and the corresponding double-stranded DNAs that contained either A, C, G, or T opposite the AP site. The homopolymeric oligodeoxynucleotide, 5′-TAMRA-(T)_5_-AP-(T)_11_-3′, was also examined as an unambiguous single-stranded DNA structure. These data demonstrated that although strand scission could occur in the context of single-stranded DNA, the double-stranded DNAs were the much preferred substrates for **C1** (Fig. 3). It was also found that the nature of the base opposite the AP site modulated the catalysis, with the rate of AP site hydrolysis being faster opposite pyrimidines than purines. These observations are consistent with the proposal that the indolinone-pyrrole subunit interacts with DNA to occupy an empty space at the AP site. Notably, the initial rate measured for the double-stranded DNA with a C opposite the AP site, was very close to the rate observed for this substrate under conditions of limiting **C1** concentration (Fig. 1f).

### Cleavage of DNA at AP sites by C1 proceeds via an intermediate involving the secondary amine

**Figure 4:**
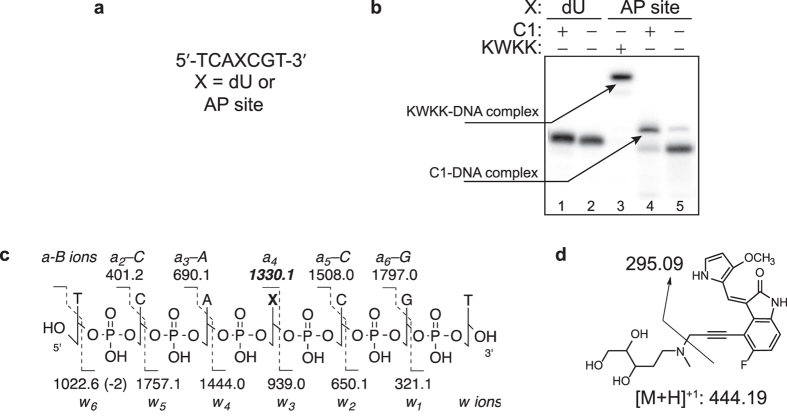
Formation of an iminium ion intermediate between **C1** and AP site. **(a)** DNA substrates. **(b)** Cyanoborohydride trapping of a complex between **C1** and AP site-containing DNA. **(c)** CID fragmentation of the reduced iminium ion intermediate. **(d)** Fragmentation of the reduced **C1**-deoxyribose conjugate after enzymatic digestion.

To test for the covalent intermediate with the ring-opened, aldehydic form of the deoxyribose, an AP site-containing ^32^P-labeled oligodeoxynucleotide (Fig. 4a) was incubated with **C1** in the presence of NaB(CN)H_3_. While this reductant slowly reacts with the AP lesion, it efficiently traps the imine- or iminium ion-conjugate. In a control reaction with the AP site-containing oligodeoxynucleotide and NaB(CN)H_3_ (Fig. 4b, lane 5), a small fraction (~3%) of the DNA manifested a decreased mobility. This low-abundance product is commonly observed in the trapping reactions^23^ and likely represents a complex with Tris molecules, as was previously shown in reactions with the malondialdehyde pyrimidopurinone DNA adduct^30^. In a positive control reaction using the lysine-tryptophan-lysine-lysine (KWKK) peptide^23^, the imine intermediate was trapped, as evidenced by the shift in DNA mobility (Fig. 4b, lane 3). In the presence of NaB(CN)H_3_ and **C1**, the majority of DNA (~80%) formed a complex that appeared as a species with decreased mobility (Fig. 4b, lane 4). The complex formation was not due to non-specific binding of **C1** to DNA, since no shift was observed when the corresponding dU-containing oligodeoxynucleotide was tested under identical conditions (Fig. 4b, lane 1). These data were consistent with the hypothesis that the reagent was able to form a covalent iminium ion intermediate, which then positions a side group for a proton abstraction from the sugar ring.

The reductive trapping of **C1** was repeated using unlabeled AP site-containing oligodeoxynucleotide (Fig. 4a), and the product was analyzed by mass spectrometry (MS). The analysis revealed a mass consistent with the reduced DNA-**C1** covalent complex (*m/z* 1134.64 for [M-2H]^−2^). Collision induced dissociation (CID) of this ion resulted in a complete set of a–B (Base) and w ions, consistent with the reduced iminium ion intermediate between **C1** and the AP site (Supplementary Figure S3). In the a–B ion series, fragmentation of the C3′-O bond is normally accompanied by the neutral loss of the nucleobase. The reduced linkage between **C1** and the AP site was expected to be less labile and as a result, we observed the a_4_ (*m/z* 1330.1) ion as well as the a_4_-B (*m/z* 1005.6). The oligodeoxynucleotide was also enzymatically digested and analyzed by MS (Fig. 4c and Supplementary Figure S4). A digestion product was observed with a mass consistent with the reduced **C1**-deoxyribose conjugate (*m/z* 444.19); fragmentation of this product ion gave a daughter ion with *m/z* 295.09, that resulted from the neutral loss of N-methyl amino-2-deoxyribitol (Fig. 4d and Supplementary Figure S4). This product was identical to that prepared from the reductive amination reaction of 2-deoxyribose and **C1**. These studies demonstrate that **C1** cleaves AP site-containing DNA through covalent catalysis involving the secondary amine.

### C1 increases the thermal stability of DNA containing an analogue of an AP site

**Figure 5:**
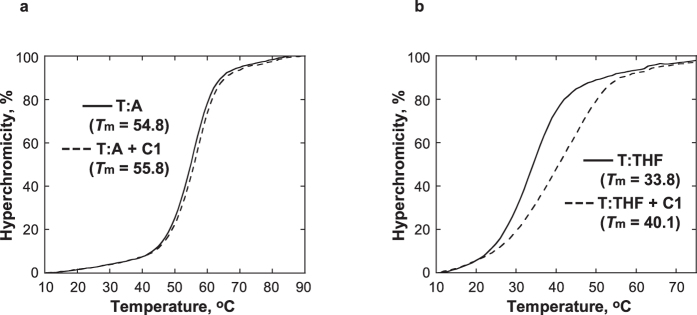
Modulation of DNA thermal stability by C1. The melting temperature curves of T:A **(a)** and T:THF **(b)** were obtained using 5 μM DNA in 10 mM sodium phosphate buffer, pH 7.0, 100 mM NaCl, and 1 mM EDTA in the absence (solid line) and presence (dashed line) of 5 μM **C1**.

The effect of **C1** was studied on thermal stability of DNA containing a tetrahydrofuran (THF), structural analogue of an AP site that is incapable of undergoing the β-elimination reaction^31^. The *T*_m_ of the duplex DNA containing THF opposite T (T:THF) increased by 6.3 °C upon addition of 1 equivalent of **C1**, while the *T*_m_ of the control DNA containing a T:A pair was less affected and only increased by 1 °C (Fig. 5). While the melting curve of T:THF with **C1** indicated thermal stabilization by **C1**, the presence of **C1** also broadened the melting curve, indicating a less cooperative melting transition. Collectively, these observations suggest that **C1** specifically binds to DNA at the AP site providing localized stabilization to the DNA helix. This is consistent with the proposed model of interactions between **C1** and DNA in which the indolinone-pyrrole moiety occupies the space available at the AP site.

### C1 has a higher affinity for DNA containing an AP site

**Figure 6:**
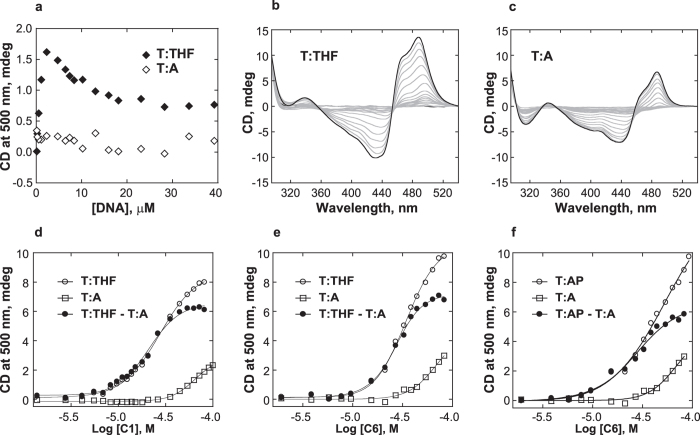
CD analyses of the interactions of **C1** and its structural analogue **C6** with duplex DNA. **(a)** Titration curves of ICD intensity at 500 nm using a constant **C1** concentration (20 μM) and increasing concentrations of DNA (reverse titration). **(b)** CD spectra for T:THF and **(c)** T:A using a constant DNA concentration (10 μM) and increasing concentrations of **C1** (forward titration). **(d)** Titration curves of ICD intensity at 500 nm versus the logarithm of the **C1** concentration for T:THF and T:A (derived from the data in panels b and c). **(e)** Titration curves of ICD intensity at 500 nm versus the logarithm of the **C6** concentration for T:THF and T:A (derived from the data in Supplementary Figure S6). **(d)** Titration curves of ICD intensity at 500 nm versus the logarithm of the **C6** concentration for T:AP and T:A (derived from the data in Supplementary Figure S6).

In order to examine the binding mode and affinity of **C1** to AP site-containing DNA, circular dichroism (CD) analyses were conducted using both THF-containing and control T:A duplex oligodeoxynucleotides (Fig. 6). The induced CD (ICD) of **C1** upon interaction with T:THF duplex was observed as a strong exciton signal, a bisignate shape with positive and negative bands relative to the absorption maximum of the free **C1**. This is generally indicative of the formation of dimeric or higher order complexes, either in a groove-binding or an external stacking-binding mode^32^. The titration of **C1** with increasing T:THF concentrations (Fig. 6a and Supplementary Figure S5) revealed both non-specific and specific interactions. At the start of titration, **C1** is in excess and non-specific interactions are favored. Upon addition of T:THF, the ICD at ~488 nm increased. At approximately 2 μM DNA concentration, the ICD band shifted to lower energy (~495 nm) and then decreased until the equivalence point and plateaued in excess of DNA indicating a specific interaction. Titration of **C1** with the control T:A DNA (Fig. 6a and Supplementary Figure S5) did not exhibit the positive ICD around 495 nm indicating the absence of specific interactions. The forward titration of T:THF also exhibited evidence of non-specific and specific interactions (Fig. 6b). At low concentrations of **C1** with DNA in excess, the ICD band was centered at ~495 nm (specific); as the concentration of **C1** increased relative to DNA, the ICD intensity increased and shifted to higher energy (~488 nm), indicative of non-specific binding as expected at high ligand concentrations. The observed hypsochromic shift may indicate a change in or a more defined conformation of **C1** when specifically bound to the AP site. Titration of the control DNA without an AP site also showed an ICD and evidence of non-specific binding (Fig. 6c). It is worth noting that the band at 450–520 nm only appeared at a 3-fold excess of **C1** with respect to DNA, making this wavelength range the best choice to construct the binding curves. Binding isotherms were constructed and analyzed using a simple bimolecular binding model as published previously^32^. The nonlinear equations that resulted from the 500 nm isotherms (Fig. 6d) gave the dissociation constant (*K*_D_) of 64 μM for the control T:A DNA (non-specific binding) and 29 μM for the T:THF DNA (specific binding); the *K*_D_ of **C1** with THF-containing duplex was calculated to be 22 μM after subtraction to correct for the non-specific binding contribution and possible ligand-ligand interactions (Table 1). The *K*_D_ values calculated at 495 nm were only slightly outside the errors of those calculated at 500 nm (Table 1).

Further, the possibility has been considered that the affinity of **C1** could be different for DNA containing a natural AP site instead of THF. To address this question, we tested structurally related but inactive compound **C6** (Fig. 2) in CD analyses with DNA containing either THF or an AP site that was created from dU by UDG treatment. The CD spectra observed for **C6** in forward titration experiments using the T:THF and T:A DNAs (Supplementary Figure S6) were generally similar to that observed for **C1** (Fig. 6b,c). The binding isotherms were constructed (Fig. 6e) and analyzed as above. The *K*_D_ of **C6** with THF-containing duplex, corrected for the non-specific interactions was calculated to be 28 μM (Table 1), which is only ~25% higher than the corresponding *K*_D_ calculated for **C1**. Thus, **C6** seems to be an appropriate model for studying interactions of **C1** with DNA. Forward titration of **C6** was then performed using DNA that contained the UDG-derived AP site (Supplementary Figure S6). Analyses of binding isotherms revealed that the affinity of **C6** to this DNA was essentially identical to THF-containing DNA (Fig. 6f and Table 1). Considering that deoxyribose at a natural AP site predominantly exists in the ring-closed, THF-like form^31^, the latter result was not surprising. It can be anticipated that prior to formation of a covalent intermediate, the affinity of **C1** to AP site-containing DNA would be comparable to that measured for THF-containing DNA. Thus, the stronger binding of **C1** to DNA containing an AP site compared to the control DNA was consistent with the thermal stability data and together supports the proposal that the indolinone-pyrrole subunit occupies an empty space at the AP site of DNA. It is likely that the affinity of **C1** for the AP site contributes to its lyase activity and provides a clear advantage over **C2**, **CS1**, **CS2**, and **CS3**, which lack the indolinone-pyrrole moiety (Fig. 2 and Supplementary Figure S2).

### Conclusions

The AP DNA strand scission catalysts described in this study represent a new class of compounds that can be utilized to cleave AP sites under physiological conditions. Based on this core structure, molecules can be designed to improve selectivity of AP site incision opposite different bases and potentially for different sequence contexts. Due to recent progress in the development of high-throughput screening techniques, these compounds could be rapidly advanced to be efficient reagents to cleave AP sites that are created through depurination or the BER pathway in cells and organisms. The optimized versions of **C1** may also have therapeutic applications, particularly in combination with many common anticancer drugs that either damage DNA, such as alkylating agents^33^, or target DNA repair, such as PARP^34^,^35^ or AP endonuclease^36^ inhibitors. It is worth mentioning that the 3′ ends created via β- or β,δ-elimination cannot be utilized by DNA polymerases without prior repair^37^. Biological consequences of the conversion of AP sites into such DNA strand breaks are expected to be complex and vary depending on the cellular capacity to repair or tolerate different types of DNA damage. The possible outcomes may include an increased therapeutic efficacy of anti-cancer treatments (more efficient cell killing) and diminished drug-induced mutagenesis. The cleavage of DNA at AP sites may be particularly beneficial for the treatment of cancers that have defects in mechanisms for repair of DNA strand breaks, such as BRCA deficient cancers^34^,^35^.

## Methods

### Materials

T4 polynucleotide kinase and UDG were purchased from New England Biolabs Inc. (Ipswich, MA). His-tagged human NEIL1 was purified according to a previously developed method^38^. His-tagged human OGG1 was purified by the same method as described for hNEIL1 using a DNA construct that was kindly provided by Dr. Gregory Verdine (Harvard University, Cambridge, MA).

The small molecule compounds **C1**, **C3**-**C7**, and **CS4**-**CS8** were provided by F. Hoffmann-La Roche Inc. The amines lacking the indolinone-pyrrole moiety **C2** and **CS1**-**CS3** were purchased from Sigma-Aldrich (St. Louis, MO). The compounds were dissolved in DMSO at concentrations ranging from 5 mM to 50 mM and stored at −20 °C.

An oligodeoxynucleotide containing an internal 8-oxo-dG and a TAMRA fluorophore on the 5′-end (5′-TAMRA-TCACC(8-oxo-dG)TCGTACGACTC-3′) was synthesized and purified in-house using previously described methodology^39^. All other oligodeoxynucleotides were purchased from Integrated DNA Technologies (Coralville, IA). Oligodeoxynucleotides containing an internal dU and a TAMRA fluorophore on the 5′-end (5′-TAMRA-TCACC(dU)TCGTACGACTC-3′ and 5′-TAMRA-(T)_5_(dU)(T)_11_-3′) or an internal tetrahydrofuran (THF) (5′-TCACC(THF)GTCGTA-3′) or dU (5′-TCACC(dU)GTCGTA-3′) were HPLC-purified by the manufacturer.

### DNA cleavage assays

To create double-stranded DNA substrates for the incision reactions, lesion-containing TAMRA-labeled oligodeoxynucleotides were combined with the corresponding complementary oligodeoxynucleotides in a buffer composed of 20 mM Tris-HCl, pH 7.5, and 100 mM KCl, and the mixtures were heated for 2 min at 90 °C and slowly cooled to 4 °C. The AP sites were created by incubating the dU-containing double-stranded DNA substrates (2.5 μM) with UDG (0.5 units/μl) for 30 min at 37 °C. The cleavage reactions were conducted with 250 nM double-stranded DNA substrates in 20 mM Tris-HCl, pH 7.5, 100 mM KCl, 0.1% (w/v) bovine serum albumin, and 0.01% (v/v) Tween-20. Concentrations of DNA glycosylases, the KWKK peptide (Sigma-Genosys), spermine (Sigma-Aldrich), **C1**, and related compounds are given in the figures or figure legends. Following incubation at 37 °C for 30 min, four volumes of formamide were added to the reactions, and DNAs were resolved through a 15% polyacrylamide gel in the presence of 8 M urea. To measure the initial rates of incision, aliquots were collected at time points that were empirically determined to be in the linear range of the product formation. The gel images were captured with the FluorChem M system (Protein Simple) using a 534 nm LED light source and 593 nm emission filter. The intensities of DNA bands were measured using Image Studio^TM^ Lite version 4.0 software (LI-COR). The data were plotted and analyzed using KaleidaGraph 4.1 software (Synergy Software).

### Trapping reactions

To prepare radioactively-labeled AP site-containing DNA for the trapping reactions, the corresponding dU-containing oligodeoxynucleotide 5′-TCA(dU)CGT-3′ (10 μM) was incubated with T4 polynucleotide kinase (1 unit/μl) in the presence of [γ-^32^P]-ATP (6000 Ci/mmol, PerkinElmer, Inc.) for 1 h at 37 °C, and AP sites were generated as described above. To test for the formation of the imine- or iminium ion-mediated complexes, ^32^P-labeled dU- or AP site-containing oligodeoxynucleotides (250 nM) were incubated with **C1** (10 μM) or KWKK (2 mM) in 100 mM Hepes-K, pH 7.3, and 50 mM NaB(CN)H_3_ at 18 °C overnight. Reactions were terminated by the addition of NaBH_4_ (100 mM final). DNAs were passed through P-6 Bio-Spin columns (Bio-Rad, Hercules, CA), mixed with an equal volume of a gel-loading solution [95% (v/v) formamide, 20 mM EDTA, 0.02% (w/v) bromophenol blue, and 0.02% (w/v) xylene cyanol], and resolved through a 20% polyacrylamide gel in the presence of 8 M urea. Radioactively-labeled DNAs were visualized using a PhosphorImager screen (GE Healthcare).

Preparation of the DNA-**C1** complexes for analyses by MS was done by incubation of dU-containing oligodeoxynucleotide (40 μM) with UDG (0.5 units/μl) for 2 h at 37 °C and subsequent reaction of DNA (16 nmoles) with **C1** (640 nmoles) in the presence of 50 mM NaB(CN)H_3_ in 100 mM Hepes-K, pH 7.3, at 37 °C overnight. An authentic standard of the reduced **C1**-deoxyribose conjugate was prepared as follows: **C1** (400 μg) and deoxyribose (2.8 mg) were dissolved in 200 μl of 50 mM potassium phosphate buffer, pH 7.0, and the reaction was stirred at 37 °C overnight. NaB(CN)H_3_ was added to reach a concentration of 50 mM, and the reaction was stirred for 3 h at 37 °C. The reaction was analyzed utilizing electrospray ionization (ESI) - liquid chromatography (LC) - MS monitoring *m/z* 444.2 corresponding to the desired product.

### Mass spectrometry

MS analyses were performed in the Vanderbilt University facility on a Waters Acquity UPLC system (Waters, Milford, MA) connected to a Finnigan LTQ mass spectrometer (ThermoElectron) equipped with an Ion Max API source and a standard electrospray probe using a Phenomenex Luna column (1 μm, 1.0 mm × 100 mm). LC conditions were as follows: buffer A contained 10 mM NH_4_CH_3_CO_2_ and buffer B contained CH_3_CN. The following gradient program was used with a flow rate of 70 μl/min: isocratic at 100% A for 1 min then: 1 to 4 min, linear gradient from 100% A to 95% A/5% B (v/v); 4 to 6 min, linear gradient to 80% A/20% B (v/v); 6 to 7 min, linear gradient to 70% A/30% B (v/v); 7 to 8 min, linear gradient to 60% A/40% B (v/v); 8–9 min, linear gradient to 50% A/50% B (v/v); 9 to 13 min, linear gradient to 100% B; 13 to 15 min, linear gradient to 100% A; 15 to 16 min, isocratic at 100% A, isocratic at 100% A from 16 to 18 min.

The temperature of the column was maintained at 50 °C and the samples (15 μl) were infused with an auto-sampler. The electrospray conditions were as follows: source voltage 4 kV, source current 100 μA, N_2_ was used as the auxiliary gas and the flow-rate setting was 20, sweep gas flow-rate setting 5, sheath gas flow setting 34, capillary voltage −49 V, capillary temperature 350 °C, and tube lens voltage −18 V. No CID offset was employed. MS-MS conditions were as follows: normalized collision energy 25%, activation *Q* 0.250, and activation time 30 ms. The isolation width in MS-MS was 2 Da. The automatic gain control settings in full MS and MS^n^ were 10000. The maximum injection time in full MS and MS^n^ were 10 ms and 40 ms, respectively. The MS data were acquired in negative mode. The number of μscan used for data acquisition in full MS and MS^n^ modes was 2 and 1, respectively. Product ion spectra were acquired over the range *m*/*z* 200–2000. The ions were selected for CID analysis and the elucidation of the CID fragmentations of the candidate oligodeoxynucleotide sequence was done with the aid of the Mongo Oligo Mass Calculator (v. 2.06) from The RNA Institute, State University of New York at Albany (http://mods.rna.albany.edu/Masspec-Toolbox). After the oligodeoxynucleotide sequence was identified, the proposed sequence was purchased from Midland Certified Reagents (Midland, TX) and subjected to the same LC-ESI-MS-MS analysis in order to compare the CID spectra.

LC-ESI-MS of the enzymatic digestion reactions was performed on an Acquity ultra-performance liquid chromatography system interfaced to a Finnigan LTQ mass spectrometer operating in ESI positive mode and using a a Phenomenex Luna column C18 (1 mm × 250 mm). The following gradient program was used with a flow rate of 60 μl/min: A: 10 mM NH_4_COOH, B: Acetonitrile, temperature of the column 50 °C; from 0 to 10 min linear gradient from 100% A to 20% A/80% B (v/v); from 10 min to 14 min linear gradient to 100% B; from 14 min to 17 min isocratic at 100% B; from 17 min to 20 min linear gradient to 100% A.

### DNA melting

The DNA melting curves were obtained using a Varian Cary400 spectrophotometer equipped with a programmable heating block. Double-stranded DNAs (5 μM) containing either THF opposite T or a control A:T pair were prepared in 10 mM sodium phosphate buffer (pH 7.0), 100 mM NaCl, and 1 mM EDTA by heating the solution at 95 °C for 5 min then cooling to 15 °C at a rate of 1 °C/min. The melting curves were determined in the absence or presence of 1 equivalent of **C1**. The UV absorption at 260 nM was monitored as a function of temperature. The temperature was increased at a rate of 1 °C/min from 10 to 90 °C. The melting temperature (*T*_m_) was determined as the inflection point of a sigmoidal function used to fit the melting curve: y = A_f_ + [(A_i_ − A_f_)/(1 + exp((x − x_0_)/dx)], where A_i_ is the initial percent hyperchromicity value or low temperature horizontal asymptote when DNA is in the double-stranded state, A_f_ is the final percent hyperchromicity value or high horizontal asymptote when DNA is in the melted state, x_0_ is the point of inflection, and dx is the change in temperature corresponding to the most significant change in percent hyperchromicity. The data were fitted using KaleidaGraph 4.0 software (Synergy Software).

### Circular dichroism

Double-stranded DNAs containing either THF or A opposite T were prepared in 10 mM sodium phosphate buffer (pH 7.0), 100 mM NaCl and 1 mM EDTA as described above. To prepare corresponding AP site-containing DNA, the dU-containing oligodeoxynucleotide was reacted with UDG (0.5 unit/μl of reaction) for 2 h at 37 °C. UDG was removed utilizing a Vivaspin centrifugal concentrator, MWCO 20 K (Sigma), and the filtrate was lyophilized. DNA was reconstituted at a final concentration of 10 μM in the buffer described above and annealed with the complement strand by heating solution at 75 °C for 2 min and slow cooling to room temperature.

The CD titrations were performed on Jasco J-810 spectropolarimeter equipped with a thermoelectrically controlled, single-cell holder. CD spectra were collected using bandwidth 1 nm, response time 0.25 s, speed 200 nm/min, sensitivity 100 mdeg, scan accumulation 5–7, and temperature of 15 °C. Binding constants were determined by performing nonlinear least squares fitting^32^. The λ selected to construct binding isotherms (500 nm) was where the contribution of non-specific binding was not significant and where DNA did not contribute to the observed signal. The data were therefore treated as a simple bimolecular binding reaction, where the CD signal versus the logarithm of total ligand concentration was plotted and a sigmoid fit (similar to equation used for *T*_m_ determination) was used to determine the dissociation constant. The reverse titration where **C1** was titrated with DNA showed a more complex binding interaction and were treated as the formalism described by Drake^40^. The data were fitted using KaleidaGraph 4.0 software (Synergy Software).

## Additional Information

**How to cite this article**: Minko, I. G. *et al.* Catalysts of DNA Strand Cleavage at Apurinic/Apyrimidinic Sites. *Sci. Rep.* **6**, 28894; doi: 10.1038/srep28894 (2016).

## Supplementary Material

Supplementary Information

## Figures and Tables

**Table 1:** Dissociation constants (*K*_D_, μM) of C1 and C6.

Compound	Wavelength	DNA				
		T:A	T:THF	T:THF corrected for non-specific interactions	T:AP	T:AP corrected for non-specific interactions
C1	500 nm	64 ± 3	29 ± 1	22 ± 1	ND	ND
	495 nm	66 ± 2	40 ± 1	26 ± 1	ND	ND
C6	500 nm	69 ± 14	35 ± 1	28 ± 1	44 ± 5	28 ± 2
	495 nm	70 ± 14	42 ± 2	28 ± 1	62 ± 15	26 ± 3

The numbers represent the *K*_D_ values and the corresponding errors obtained from fitting the experimental data to a sigmoidal curve.
