# Supplementary material for: Catalysts of DNA Strand Cleavage at Apurinic/Apyrimidinic Sites
Source: Sci Rep. 2016 Jul 1;6:28894. doi: 10.1038/srep28894 (PMC4929455; doi:10.1038/srep28894)
Supplement: Supplementary Information [file srep28894-s1.pdf]

Catalysts of DNA Strand Cleavage at Apurinic/Apyrimidinic Sites

Irina G. Minko, Aaron C. Jacobs, Arnie R. de Leon, Francesca Gruppi, Nathan Donley, Thomas M. Harris, Carmelo J. Rizzo, Amanda K. McCullough, R. Stephen Lloyd

Supporting Information: Supplementary Figures S1-S6

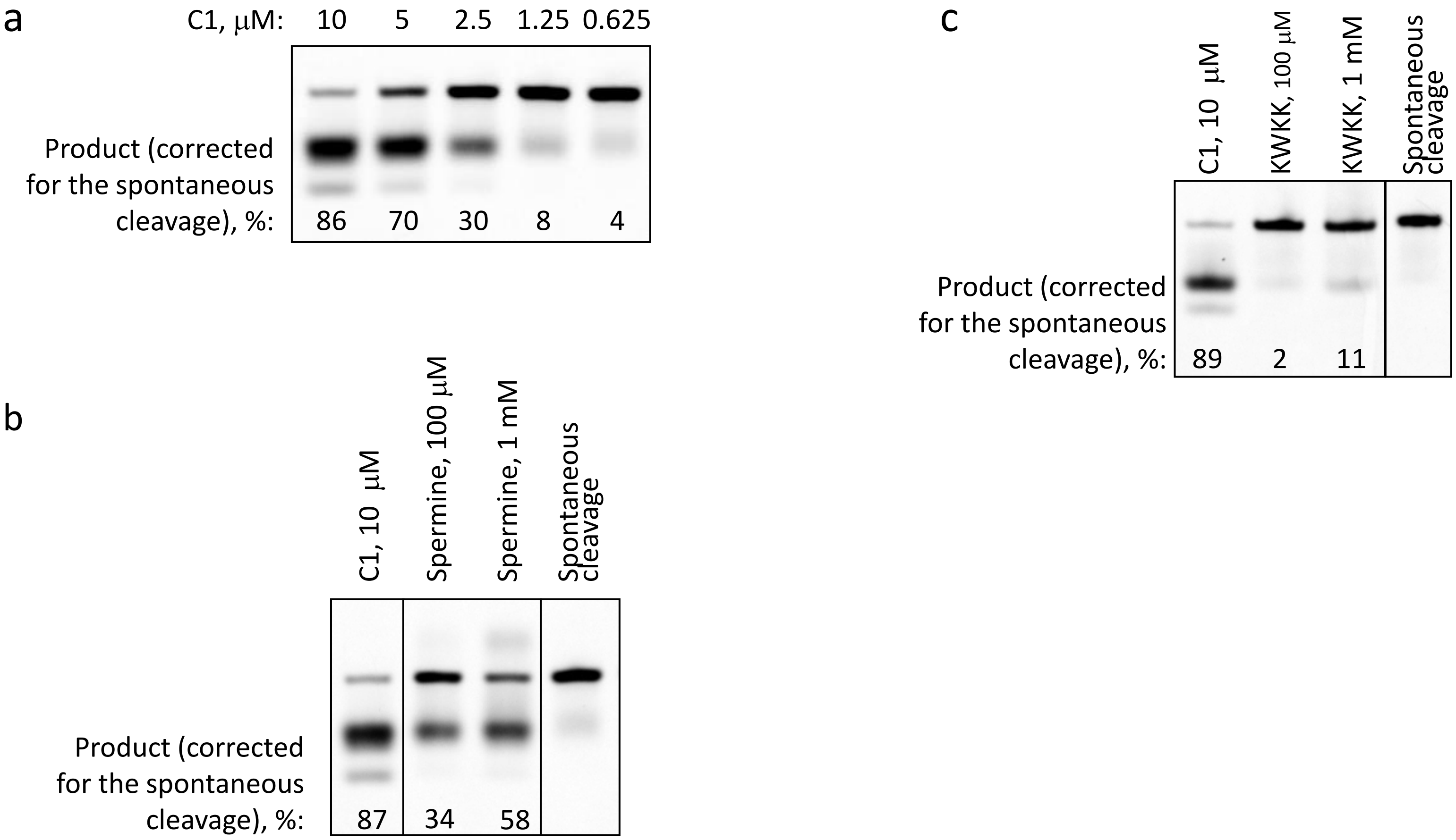

Minko et al., Supplementary Figure S1. Non-enzymatic DNA cleavage at an AP site. (a) Concentration-dependent scission of AP site-containing DNA (250 nM) by **C1**. (b) Scission of AP site-containing DNA (250 nM) by **C1** or spermine. (c) Scission of AP site-containing DNA (250 nM) by **C1** or KWKK. Reactions were carried out at 37 ^{o}C for 30 min.

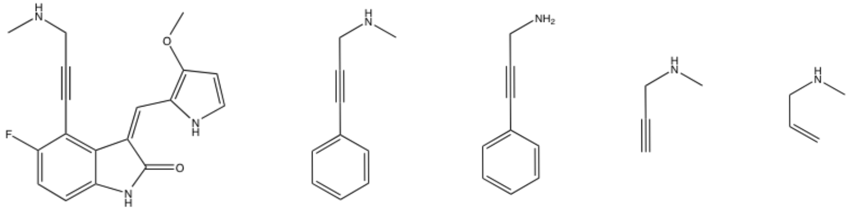

C1 C2 CS1 CS2 CS3

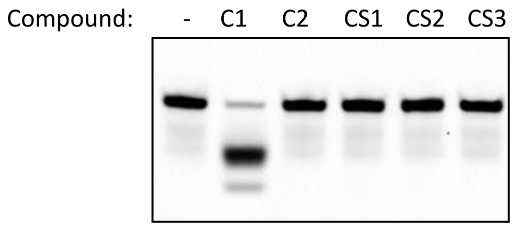

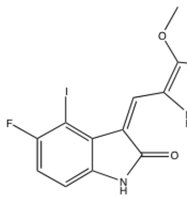

CS4

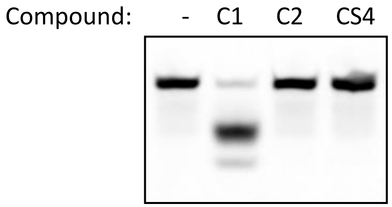

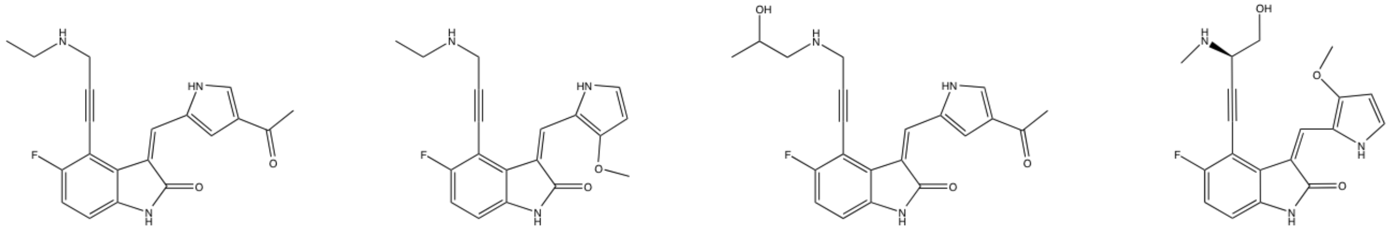

CS5 C7 CS6 CS7

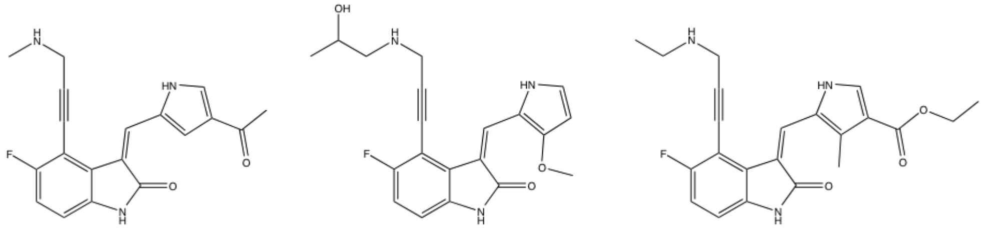

C4 CS8 C5

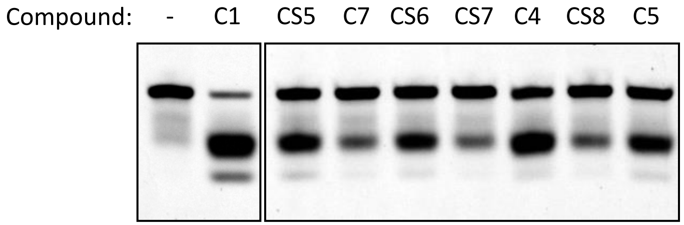

Minko et al., Supplementary Figure S2. Test for the abilities of various compounds (10 μM) to incise AP site-containing DNA (250 nM). Reactions were carried out at 37 ^{o}C for 30 min.

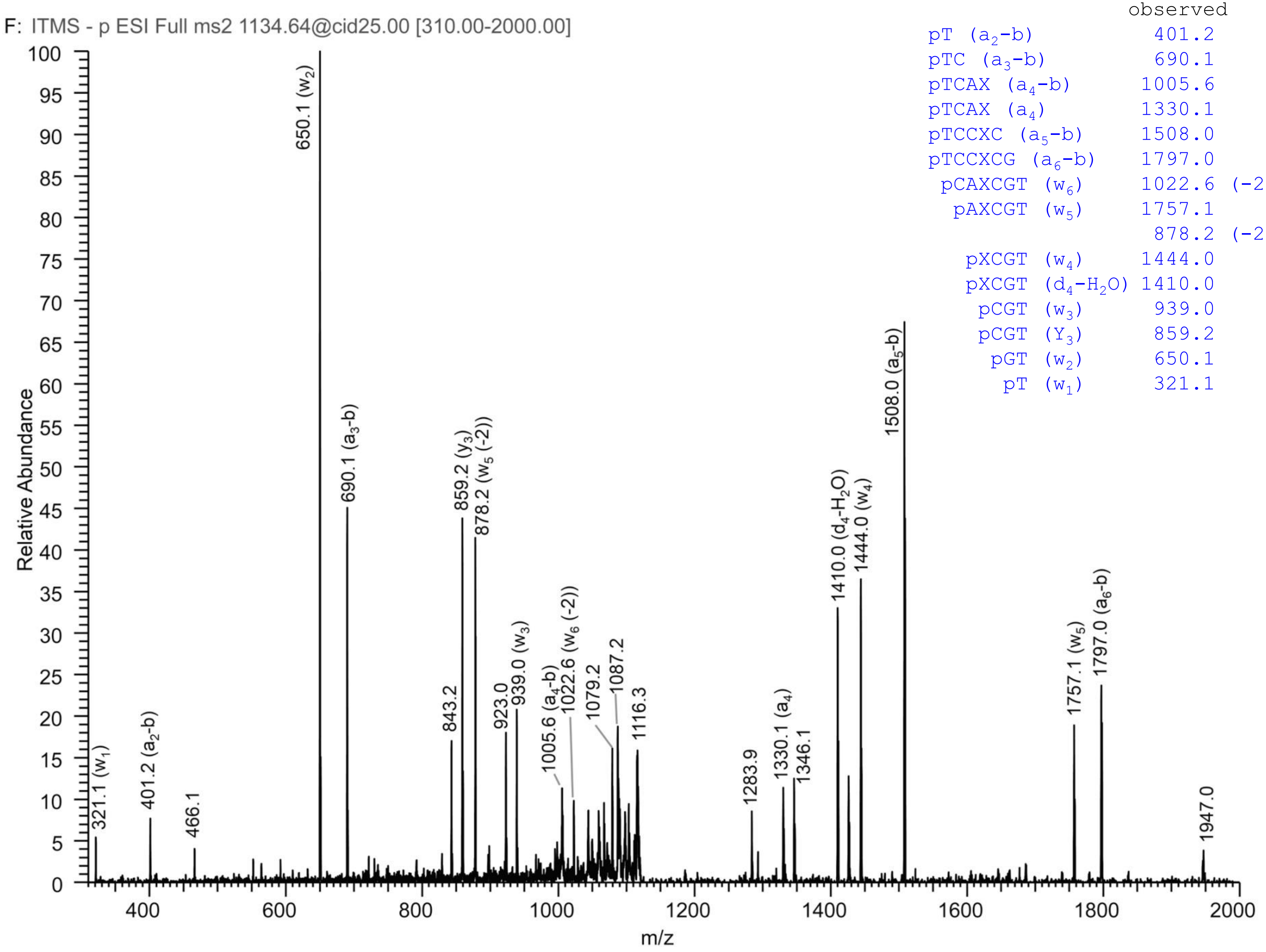

		observed	theoretical
pT	(a_2-b)	401.2	401.29
pTC	(a_3-b)	690.1	690.47
pTCAX	(a_4-b)	1005.6	1005.18
pTCAX	(a_4)	1330.1	1329.8
pTCCXC	(a_5-b)	1508.0	1507.97
pTCCXCG	(a_6-b)	1797.0	1797.16
pCAXCGT	(w_6)	1022.6 (-2)	1022.64 (-2)
pAXCGT	(w_5)	1757.1	1757.10
		878.2 (-2)	878.10 (-2)
pXCGT	(w_4)	1444.0	1444.89
pXCGT	(d_4-H_2O)	1410.0	1410.15
pCGT	(w_3)	939.0	939.59
pCGT	(Y_3)	859.2	859.61
pGT	(w_2)	650.1	650.41
pT	(w_1)	321.1	321.20

Minko et al., Supplementary Figure S3. CID fragmentation of the reduced complex between **C1** and the AP-site containing oligodeoxynucleotide 5’-TCA(AP)CGT-3’.

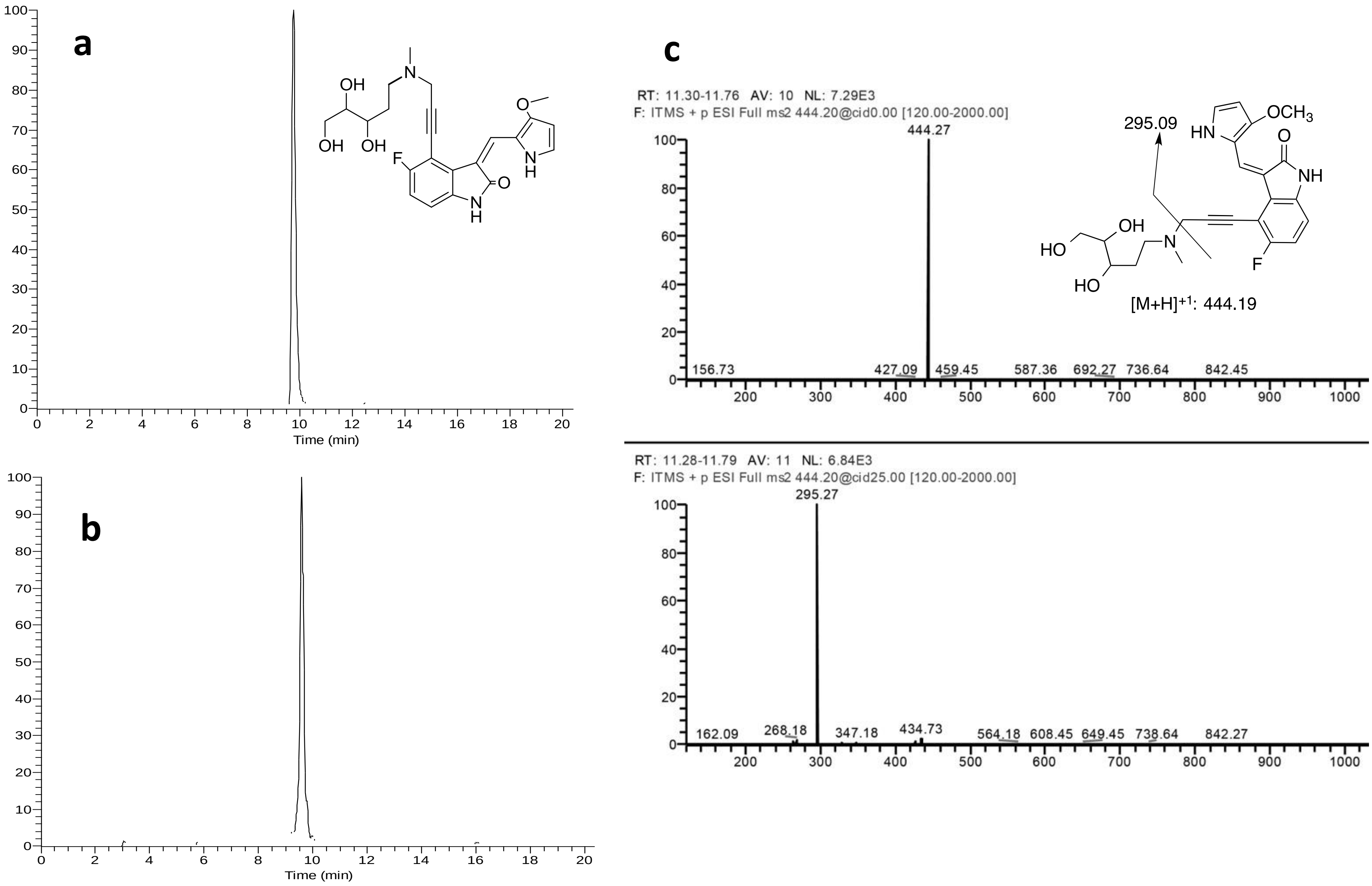

Minko et al., Supplementary Figure S4. ESI-LC-MS analysis of enzymatic digestion of the **C1**-modified oligonucleotide 5'-TCA(AP)CGT-3' . MS[2] was utilized to monitor *m/z* corresponding to **C1**-deoxyribose adduct. Panel **a** shows the reconstructed chromatogram for the **C1**-adduct standard, synthesized as reported in experimental section. Panel **b** is the reconstructed chromatogram from the enzyme digestion reaction corresponding to the reduced **C1**-deoxyribose conjugate (*m/z* 444.2) at 9.8 min. Panel **c** is the ionization profile for the peak at 9.8 min showing a characteristic fragment with *m/z* 295.2.

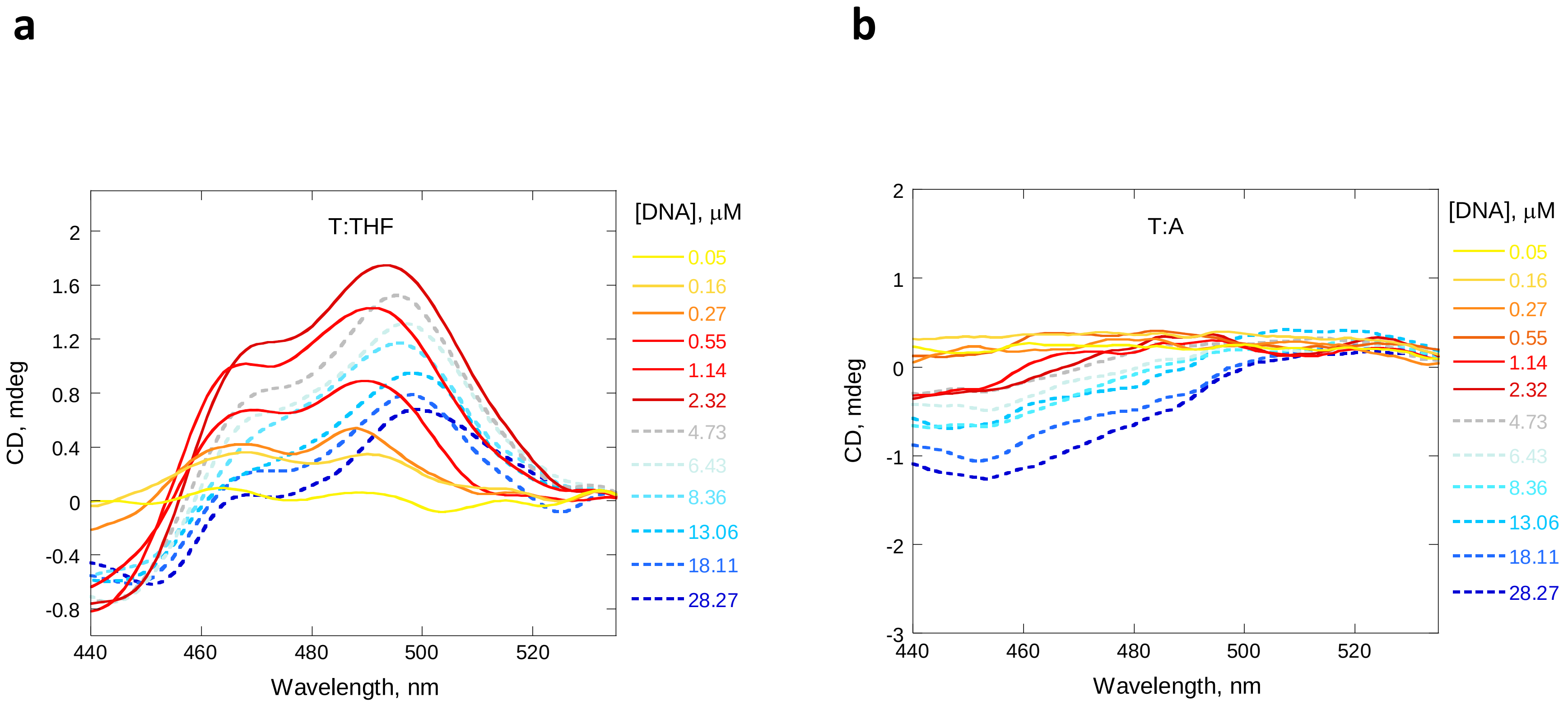

Minko et al., Supplementary Figure S5. The interaction of **C1** with DNA by CD analyses. ICD spectra of **C1** (20 μM) in the presence of increasing concentrations of T:THF (a) or T:A (b) DNA (reverse titration).

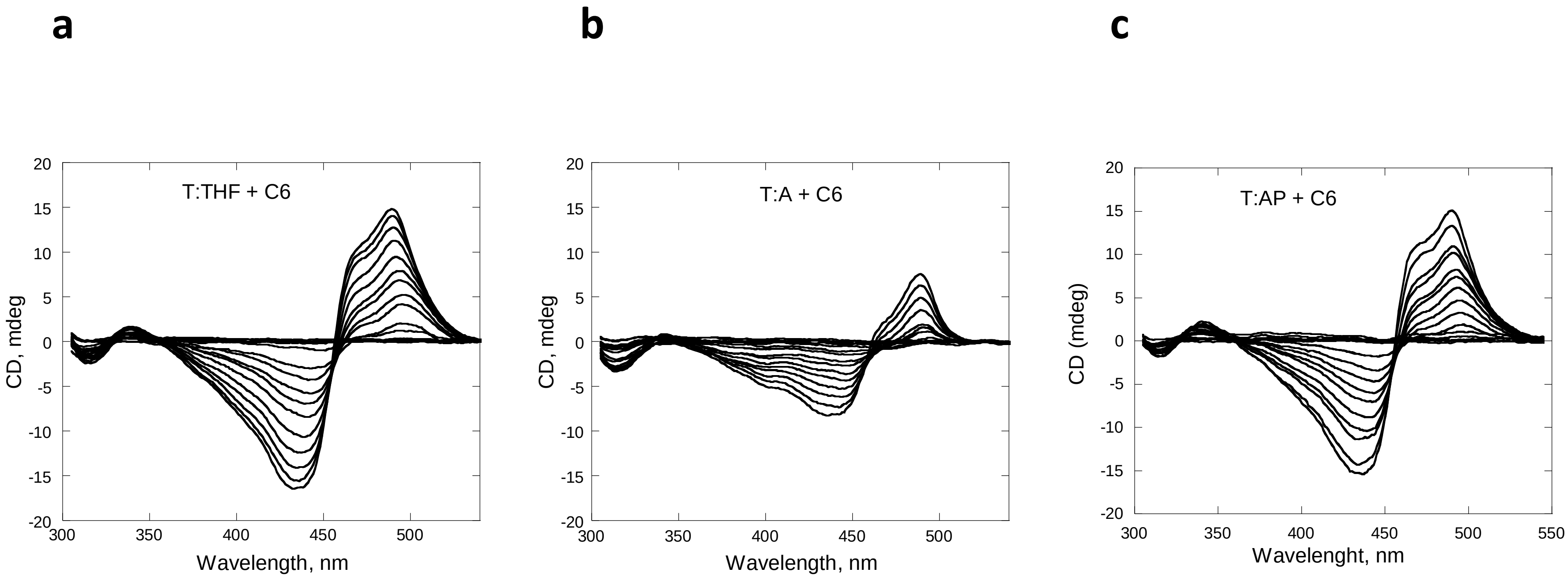

Minko et al., Supplementary Figure S6. The interaction of **C6** with DNA by CD analyses. CD spectra for T:THF (a), T:A (b), and T:AP (c) using constant DNA concentration (10 μM) and increasing concentrations of **C6** (forward titration).
